# College Student Problematic Internet Use and Digital Communication Medium Used With Parents: Cross-Sectional Study

**DOI:** 10.2196/17165

**Published:** 2020-04-23

**Authors:** Bradley Kerr, Jon D D'Angelo, Ali Diaz-Caballero, Megan A Moreno

**Affiliations:** 1 University of Wisconsin-Madison Madison, WI United States

**Keywords:** parents, young adults, social media

## Abstract

**Background:**

Problematic internet use (PIU) is associated with mental health concerns such as depression and affects more than 12% of young adults. Few studies have explored potential influences of parent–college student digital communication on college students’ risk of PIU.

**Objective:**

This study sought to understand the relationship between parent–college student digital communication frequency via phone calls, text messages, and Facebook contacts and PIU among college students.

**Methods:**

Incoming first-year students were randomly selected from registrar lists of a midwestern and northwestern university for a 5-year longitudinal study. Data from interviews conducted in summer 2014 were used. Measures included participants’ daily Facebook visits, communication frequency with parents via phone call and text message, and 3 variables related to Facebook connection status and communication: (1) parent–college student Facebook friendship status, (2) college student blocking personal Facebook content from parent, and (3) Facebook communication frequency. PIU risk was assessed using the Problematic and Risky Internet Use Screening Scale. Analysis included participants who reported visiting Facebook at least once per day. Multiple linear regression was used, followed by a post hoc mediation with Hayes process macro to further investigate predictive relationships among significant variables.

**Results:**

A total of 151 participants reported daily Facebook use and were included in analyses. Among these participants, 59.6% (90/151) were female, 62.3% (94/151) were from the midwestern university, and 78.8% (119/151) were white. Mean Facebook visits per day was 4.3 (SD 3.34). There was a collective significant effect between participant daily Facebook visits, college student–parent phone calls, texts, and all 3 Facebook connection variables (*F*_6,144_=2.60, *P*=.02, *R*^2^=.10). Phone calls, text messages, and Facebook contacts were not associated with PIU risk. However, two individual items were significant predictors for PIU: participant daily Facebook visits were positively associated with increased PIU risk (*b*=0.04, *P*=.006) and being friends with a parent on Facebook was negatively associated with PIU risk (*b*=–0.66, *P*=.008). Participant daily Facebook visits were not a significant mediator of the relationship between college student–parent Facebook friendship and PIU risk (*b*=–0.04; 95% CI –0.11 to 0.04).

**Conclusions:**

This study did not find support for a relationship between parent–college student digital communication frequency and PIU among college students. Instead, results suggested Facebook friendship may be a protective factor. Future studies should examine how a parent-child Facebook friendship might protect against PIU among children at varying developmental stages.

## Introduction

Problematic internet use (PIU) is an emerging health concern among young adults, defined as technology use that may involve impulsive and risky internet use; internet use dependency; social, physical, and emotional impairment; and psychological risks [1]. Numerous mental health concerns, including attention deficit hyperactivity disorder, social phobia, hostility, depression, and increased likelihood and severity of suicidal ideation, have been linked to PIU [2,3]. Studies suggest that college students experience PIU at rates of 4% to 22% [4-7]. These findings support the need for an understanding of influences on the development of PIU among young adult college students to inform prevention efforts.

Parents may play roles in college students’ development of PIU. Parent characteristics such as low education level, young age, and lifetime use of tobacco and illicit drugs have been linked to college students’ excessive internet use [8]. In addition, parental relations, such as those involving rejection and overprotection, are associated with increased risk for PIU [9]. Another influential factor may be parents’ own technology behaviors. Parents’ use of social networking and mobile phones has been found to predict their children’s engagement with these media [10,11]. Given the importance of both the parental relationship and parent-child technology behaviors, it is essential to understand mechanisms by which parents can exert influence over their children’s internet use, even at the young adult stage.

Technology itself may offer a vehicle for parents to influence college students’ technology use through modeling their own. According to social cognitive theory, new behaviors may originate from noting how others act [12]. Observing parents’ use of technology could therefore influence college students’ behaviors. Further, relevant cues about parents’ technology use may be transmitted through devices themselves. Parents who initiate frequent, high-volume text message exchanges with their college student children, for example, may unintentionally convey suggestions about acceptable technology use rates. This mode of conveying norms could hold particular relevance for college-attending youth, who often live away from parents and could rely on digital communication frequently. For this reason, rates of parent-child digital communication could be important to college students’ risk of PIU.

Digital communication between parents and college students is common and has increased in recent years [13]. Many college students not only allow the creation of friendship links between themselves and their parents but also use social networking sites such as Facebook to stay in touch [14]. Following the establishment of a social media link, few students report ever blocking parents from seeing individual content they have posted [15]. Increasing digital connectedness between parents and their college student children warrants exploring the effects of these changing relationships.

While parent-child digital connectedness could have an effect on PIU among college students, few studies have explored this possibility. Some evidence suggests that parental mediation around children’s use of the internet is associated with reductions in excessive internet use [16,17]. Thus, it may be that parent-child digital communication provides an opportunity for mediation and modeling of healthy internet use behaviors. However, as parent-child digital communication increases, so too may modeling of unhealthy internet use by parents [10]. Toward clarifying this relationship, this study aimed to understand the relationship between parental digital communication and PIU risk among college students. This purpose was addressed by considering possible effects of three different digital communication media: phone calls, text messages, and Facebook contacts via public timeline post or private message. Phone calls, text messages, and Facebook contacts were the focus of this study given evidence that many adults use all three media [13,18].

## Methods

### Design

This cross-sectional study used data from a 5-year, longitudinal study involving yearly phone interviews of college students starting at the time of matriculation. For this secondary analysis, phone interview data from a single time point when questions about parent communication were integrated into yearly interviews were used.

### Setting

This study took place at two universities, one midwestern and the other northwestern. Data were collected in the summer of 2014, when participants were entering their fourth year of college. The relevant institutional review boards reviewed and approved this study.

### Participants

In the summer of 2011, researchers randomly selected incoming first-year students from registrar lists of the targeted midwestern and northwestern universities. Potential participants received a postcard introducing them to the 5-year, longitudinal study, followed by four rounds of recruitment contacts by email, phone, and Facebook message. Eligible potential participants included first-year students with full-time status who owned Facebook profiles, spoke English, and did not move to campus before fall as part of an early enrollment program. For this secondary analysis study, participants were included if they completed a phone interview following their third year of college and they were at least daily users of Facebook.

### Phone Interviews

After the third year of college, participants in this study completed phone interviews. This approach allowed for data collection from participants living more than a half hour away from the study site. Phone interviews have been successfully implemented in past studies to collect sensitive health information [19,20]. Interviews were conducted at convenient times for participants and lasted 30 to 60 minutes. Participants received an incentive of $40 after the interview.

### Measures

#### Digital Communication With Parents

Interviews assessed predictor variables around frequency of communication with parents via phone calls, text messages, and Facebook contacts. To assess phone calls, researchers asked the question, “In an average week, how many times did you talk on the phone with one or both of your parents while at college?” For text messages, participants answered the questions, “In an average week, on how many days did you send or receive a text from your parents?” and “On the days that you texted with your parents, about how many texts were sent back and forth during that day/those days?”

To measure frequency of Facebook contacts, it was necessary to assess the parent-student Facebook connection status (ie, whether the student’s account was made visible to parents). To this end, participants indicated whether at least one parent or guardian owned a Facebook account and if they had at least one parent as a friend on the site. Further, participants indicated whether they blocked any content from their parent Facebook friends. Those who reported having at least one parent who was a Facebook account owner and Facebook friend were asked, “In an average week, how many times did you communicate with your parents or guardians using Facebook?” Interviewers clarified that communication via Facebook included public timeline posts or private messages.

#### Problematic Internet Use

This study assessed the outcome variable of risk of problematic internet use during the third year of college using the Problematic and Risky Internet Use Screening Scale (PRIUSS) [21]. This scale includes 18 items addressing problematic internet use. This measure has been shown to have strong reliability and content validity in the college population [22]. Participants chose a response of never, rarely, sometimes, often, or very often for each of the 18 items. Each response was assigned a numerical value from 0 (never) to 5 (very often); all items were summed. A score of 25 or greater indicated risk of problematic internet use.

#### Demographics and Facebook Use Variables

Demographic variables included age, gender, race/ethnicity, and university; Facebook use during the third year of college was also reported. Participants indicated their Facebook account ownership status and whether they visited the site daily. Those who made daily visits indicated how many times they typically did so per day.

### Analysis

Toward examining predictors among participants most likely to experience PIU, we included only participants who reported at least daily use of Facebook in analyses. Digital communication with parents, PIU, and demographic and Facebook use variables were analyzed using descriptive statistics. Multiple linear regression was used to test the predictive relationship of digital communication with parents and social media use toward PRIUSS scores. Results were considered statistically significant when *P*<.05. A post hoc mediation analysis with Hayes process macros allowed further investigation into predictive relationships among significant variables [23].

## Results

### Participants

Among the 329 participants enrolled in the larger study in the summer of 2014, 151 completed phone interviews and indicated daily use of Facebook and were thus included in analyses. These participants had an average of 4.3 (SD 3.34) Facebook visits per day. The majority were female (90/151, 59.6%), from the midwestern university (94/151, 62.3%), and white (119/151, 78.8%). A PRIUSS score of 25 or greater (indicating risk of PIU) was met by 15.9% (24/151) of participants with daily Facebook use. See Table 1 for full demographic results.

**Table 1:** Participant demographics.

Characteristic		Value, n (%)	
**Gender**			
	Female		90 (59.6)
	Male		61 (40.4)
**University**			
	Midwestern		94 (62.3)
	Northwestern		57 (37.7)
**Race/ethnicity**			
	White		119 (78.8)
	Asian		14 (9.3)
	Multiracial		8 (5.3)
	Hispanic		5 (3.3)
	African American		2 (1.3)
	East Indian		1 (0.7)
	Native American/Alaskan		1 (0.7)
	Other		1 (0.7)

### Digital Communication With Parents

Most participants (145/151, 96.0%) reported that on a weekly basis they had at least one phone call with their parents. These participants had an average of 3.0 (SD 3.5) calls with parents per week. All participants (151/151, 100%) indicated at least one weekly exchange of text messages with parents and did so an average of 3.5 (SD 2.1) days per week.

Nearly all participants (146/151, 96.7%) reported that at least one parent owned a Facebook account. Among them, the majority (136/146, 93.2%) indicated that they had at least one parent as a Facebook friend. Few (24/136, 17.6%) reported blocking Facebook content from a parent. Less than half (64/136, 47.0%) suggested they used Facebook contacts, such as a timeline post or private message, to communicate with parents. These participants reported using Facebook to interact with parents an average of 1.8 (SD 1.4) times per week.

### Associations Between Parent-Child Digital Communication and Risk of Problematic Internet Use

The multiple linear regression indicated that there was a collective significant effect between participant daily Facebook visits, participant-parent phone calls, text messages, parent Facebook friendships, weekly parent Facebook contacts, and blocking content from parents (*F*_6,144_=2.60, *P*=.02, *R*^2^=.10; model fit: *R*=.321; *R*^2^=.097; adjusted *R*^2^=.06). Weekly phone calls, text messages, and Facebook contacts were not associated with risk of PIU. The number of daily Facebook visits was found to be associated with increased risk of PIU (*P*=.006), while a parent Facebook friendship was associated with decreased PIU risk (*P*=.008). Weekly parent phone calls, text messages, and Facebook contacts were not significantly associated with risk of PIU. See Table 2 for full multiple linear regression results.

**Table 2:** Predictors of risk of problematic internet use.

Characteristics	B	SE B	β	*t* value	*P* value
Number of daily Facebook visits	0.036	0.013	0.223	2.808	.006
Weekly parent phone calls	–0.006	0.012	–0.041	–0.517	.61
Weekly parent text messages	–0.008	0.010	–0.066	–0.826	.41
Parent Facebook friendship	–0.661	0.244	–0.219	–2.708	.008
Weekly parent Facebook contacts	0.003	0.034	0.007	0.093	.93
Blocking Facebook content from parents	–0.101	0.121	-0.006	–0.079	.94

The post hoc mediation analysis with Hayes process macro did not show the number of daily Facebook visits to be a significant mediator between parent Facebook friendship and PIU risk (*b*=–0.04; 95% CI –0.11 to 0.04).

## Discussion

### Principal Findings

The purpose of this cross-sectional, 2-site phone interview study was to understand the relationship between parent–college student digital communication and PIU among these young adults. Findings did not support digital communication—via parent-child phone calls, text messages, and Facebook contacts—as a risk factor for PIU. Further, the study suggested parent–college student Facebook friendships are associated with decreased risk of PIU among college students who use Facebook daily and that text messages and phone calls are commonly used digital communication media.

In this study, parent–college student phone calls, text messages, and Facebook contacts were not associated with college students’ risk of PIU. Thus, support was not found for the possibility that parents convey norms of excessive technology use when they communicate with their college student children on digital platforms at high rates. Instead, it may be that the immersive nature of content exchanged through digital media affects the student’s risk of PIU. If a peer sends their friend a meme via text message, for example, the recipient may be prompted to find additional content to share in return, thereby engaging with the internet further. Meanwhile, if parents primarily contact their children for practical purposes, such as coordinating plans, interactions with them may not motivate additional phone and internet use. This explanation is consistent with an emerging body of literature suggesting that the manner of engagement with social media, not just the duration, is associated with well-being [24-26].

An unexpected, additional finding was that Facebook friendships with parents were associated with decreased risk of PIU and that the number of daily Facebook visits among college students who used Facebook daily did not mediate this relationship. It is also worth noting that a large majority of participants were Facebook friends with at least one parent. Thus, results did not yield evidence that a parent’s online presence leads to lower engagement with social media on the part of the many college students who have parents as Facebook friends. Instead, it may be that a parent–college student Facebook friendship allows mediating and modeling of safe, healthy internet use. Another possible reason for this finding is that some aspects of the parent–college student relationship affect both these young adults’ likelihood of being Facebook friends with their parents and having increased risk of PIU. This possibility is consistent with previous studies suggesting a connection between parenting factors, such as parenting and attachment style, and adolescents’ and young adults’ use of mobile phones [27-32]. Similarly, college students have reported ways that social media connection benefits their relationship with parents, including sharing important life events and memories [14]. Thus, an additional possibility is that Facebook friendship with parents could decrease risk of PIU in college students by improving parental relationships. While the reason for the association between Facebook friendships with parents and college students’ reduced risk of PIU remains unclear, this finding provides additional support for the importance of parents, and their potential protective effect, in college students’ development of PIU.

An important third finding was that participants frequently communicated with parents via text messages and phone calls but seldom via Facebook contacts. This finding is consistent with a previous study showing text messages to be among the most frequently used parent–young adult communication media and suggesting that adopting a new platform for family communication requires the development of technologies that improve how families communicate with each other [33]. It may be that college students and their parents find text messages and phone calls adequate for the nature of their digital communications, if interaction often serves practical purposes such as coordinating plans. Further, compared with other media, texting message and phone call platforms may be viewed as simple, infrequently updated, and easy to use.

### Limitations

This study has limitations to consider. Data were collected from large state universities. It is not clear whether findings generalize to other institution types such as small private colleges. Nevertheless, our study population included two geographically diverse schools, and participants had similar demographic makeup to that of the involved universities. Further, participants were predominantly white. Since parent-child relationships occur in differing cultural contexts [34-37], it is not clear whether findings generalize to nonwhite families. In addition, in this cross-sectional study, the direction of significant relationships found is unknown. There is a need for culturally specific research investigating causal pathways between parent–college student digital communication and college students’ risk of PIU. Finally, this study’s self-reported findings may be subject to recall bias. Future studies may use ecological momentary assessment approaches to circumvent this limitation.

### Conclusion

This study did not find evidence that digital communication with parents is a risk factor for PIU among college students. Instead, findings suggest a possibility that social media connections between parents and college students may be protective. Implications are relevant to guidelines that pediatricians may offer to parents regarding digital communication with their children. It may not be necessary to advise limiting use of digital communication with children, particularly among the many parents who have already established social media connections with their children. In fact, parents should be informed of potential benefits of social media connection.

Additional research is needed toward developing guidelines for parents around digital communication with their children. Future studies should examine the role that a parent-child Facebook friendship may play in protecting against PIU among college students, such as allowing modeling of healthy internet use by parents or promoting regular contact and therefore supportive relationships and well-being. Further, investigations into risks and benefits of social media interactions among parents and children across development stages are important to inform guidelines for parents on this topic. What protects against PIU at one developmental stage may not at another. A third important area to consider is the nature of content shared between parents and children in digital communications. Meme sharing, coordination of plans, and serious conversations facilitated by digital communication may have differing effects on their recipients. These future inquiries may support parents in using digital communication as a tool to promote their children’s well-being.

## Abbreviations

PIU: problematic internet use
PRIUSS: Problematic and Risky Internet Use Screening Scale
